# Combined targeting of TCF7L1/2, PTEN, CDK6, and BCCIP by microRNA miR‐29c‐3p is associated with reduced invasion and proliferation of endometriotic cells

**DOI:** 10.1002/rmb2.12645

**Published:** 2025-03-25

**Authors:** Teresa Helene Wentges, Heba M. El‐Shorafa, Janine Beckmann, Michael Gabriel, Matti Poutanen, Burkhard Greve, Ludwig Kiesel, Sebastian D. Schäfer, Martin Götte

**Affiliations:** ^1^ Department of Gynecology and Obstetrics Münster University Hospital, Research Laboratory Münster Germany; ^2^ Department of Laboratory Medical Sciences, Faculty of Medical Sciences Alaqsa University Gaza Palestine; ^3^ Department of Obstetrics and Gynecology Institute of Medicine, University of Turku Turku Finland; ^4^ Research Centre for Integrative Physiology and Pharmacology Institute of Biomedicine, University of Turku Turku Finland; ^5^ Department of Radiotherapy‐Radiooncology Münster University Hospital Münster Germany; ^6^ Department of Gynecology and Obstetrics Clemenshospital Münster Münster Germany; ^7^ Cells‐in‐Motion Interfaculty Centre (CiMIC) University of Münster Münster Germany

**Keywords:** CDK6, endometriosis, microRNA, miR‐29c, p21

## Abstract

**Purpose:**

Endometriosis is a chronic gynecological disorder associated with pain symptoms and infertility. The expression of microRNA miR‐29c‐3p is dysregulated in endometriosis. We aimed to identify novel molecular targets of miR‐29c‐3p functionally linked to proliferation and invasive growth in endometriosis.

**Methods:**

The epithelial endometriotic cell line 12Z and primary endometriotic stromal cells (PESC) were transfected with control miRNA or pre‐miR‐29c‐3p, and subjected to cell cycle analysis, cell viability, wound healing, and Matrigel invasion assays. Expression of bioinformatically predicted miR‐29c‐3p targets was analyzed by qPCR and western blot. Target gene expression in endometriotic lesions and healthy endometrium was studied in the EndometDB endometriosis database.

**Results:**

miR‐29c‐3p decreased 12Z and PESC cell viability and the proportion of PESC in the S‐phase. 12Z cell invasion, but not migration, was decreased after miR‐29c‐3p upregulation. miR‐29c‐3p decreased the mRNA expression of *CDK6*, *BCCIP*, *TCF7L1*, *TCF7L2*, *PTEN*, *COL4A1*, *E‐Cadherin*, and *N‐Cadherin*. A decrease of CDK6 and PTEN and an increase of p21 were confirmed at the protein level. EndometDB database analysis demonstrated dysregulated expression of the selected targets in both deep endometriosis and ovarian endometriosis.

**Conclusions:**

miR‐29c‐3p effectively curbs endometriotic cell proliferation and invasion by combined inhibition of cell cycle regulators and transcription factors, unveiling a promising therapeutic strategy.

## Graphical abstract

miR‐29c, a microRNA dysregulated in endometriosis, is targeting several cell cycle and transcriptional regulators, which are significantly dysregulated in endometriosis. In a cell culture model, miR‐29c induced changes in cell viability, cell cycle progression and invasive growth, which are cellular properties regulated by the newly identified molecular targets.

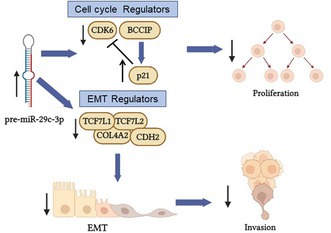

## 1 INTRODUCTION

Endometriosis is an estrogen‐dependent, chronic inflammatory disease characterized by the ectopic growth of endometrium‐like glands and stroma outside the uterus.^1^ It is the second most common benign disease in women after adenomyosis^2^ with a worldwide prevalence of 10% of women in their reproductive age.^3^ Endometriosis‐associated symptoms have serious consequences on both work ability and social satisfaction, such as dysmenorrhea, chronic pain in the lower abdomen, dysuria, and bleeding disorders.^1^, ^2^ Several hypotheses have been suggested to explain the pathogenesis of endometriosis. The most plausible one was developed by Sampson, in which endometrial cells migrate up the fallopian tubes into the abdominal cavity by retrograde menstruation and attach themselves to the peritoneal surface.^4^ In addition, obstacles to drainage in the vagina due to cervical stenosis seem to promote the development of endometriosis. This observation supports the theory of Sampson.^5^ Other theories have been proposed, including lymphatic vascular distribution,^6^ the coelomic metaplasia theory,^7^ stem cell recruitment theory,^8^, ^9^ and embryogenetic theory.^10^, ^11^

Recent studies have illustrated a dysregulated expression of several microRNAs (miRNA) in eutopic or ectopic endometrium, suggesting their potential as diagnostic markers as well as therapeutic targets.^12^, ^13^, ^14^, ^15^ miRNAs are noncoding, highly conserved ribonucleic acids of about 22 nucleotides in length. They play a crucial role in posttranscriptional gene regulation by binding to the 3′ untranslated region of the target mRNA, leading to translational inhibition or mRNA degradation.^16^, ^17^ In the female reproductive system, a dysregulated miRNA expression has been observed in uterine leiomyomas, various gynecological tumors, and endometriosis. They play a fundamental regulatory role in key cellular processes such as cell survival, matrix remodeling, proliferation, and angiogenesis.^16^, ^18^, ^19^, ^20^, ^21^ Among these miRNAs, miR‐29c‐3p is of distinctive relevance to endometriosis‐associated infertility, as it was shown by several studies to be dysregulated in endometriosis patients compared to healthy subjects.^16^, ^20^, ^21^, ^22^, ^23^, ^24^, ^25^ It was first described by Yu et al. in leukemia cell lines.^26^ Since then, it has been studied in various diseases, mainly tumors. For instance, downregulation of miR‐29c was observed in nasopharyngeal carcinomas,^27^ pancreatic carcinoma,^17^ and lung squamous cell carcinoma.^28^ These data suggest its role in invasion, proliferation, and metastatic potential with an influence on various signaling pathways. Moreover, several studies on gynecological diseases are showing a downregulation of miR‐29c in ovarian carcinomas resulting in promoted metastasis and poor prognosis. A reduced expression of miR‐29c was detected in leiomyomas of the uterus, which is associated with decreased cell proliferation and motility.^18^

Although miR‐29c‐3p is dysregulated in endometriosis, only a few functional studies were performed in this context. For instance, the study of Long et al. employed CRL‐7566 endometriosis cells, which were established from benign ovarian cysts obtained from endometriosis patients. The cells were employed as a model of ovarian endometriosis cells to study the functions of miR‐29c‐3p, demonstrating a c‐Jun‐dependent impact on cell proliferation and invasion.^23^ Additional studies demonstrated a regulation of the proangiogenic cytokine VEGF‐A by miR‐29c‐3p in endometriotic stroma cells,^29^ while miR‐29c‐3p‐dependent regulation of FKBP4 has been linked to progesterone resistance in a baboon model of endometriosis.^24^

Since microRNAs have multiple targets, and since they can exert effects in a cell‐type‐dependent manner,^30^, ^31^ the aim of this study was to identify the novel molecular targets of miR‐29c‐3p functionally linked to proliferation and invasive growth in endometriosis. We further aimed to identify novel targets of miR‐29c‐3p linked to aberrant proliferation and invasion, and to confirm the dysregulation of these targets in endometriotic tissue utilizing the Turku EndometDB database.^32^

Hence, we utilized the 12Z, which were originated from endometrial tissue located on the abdominal lining (peritoneum) of a 37‐year‐old female endometriosis patient undergoing laparoscopy.^33^ Additionally, we used primary endometriotic stromal cells (PESC) isolated from rectovaginal septum (PESC1), uterine serosa (PESC2), and peritoneal (PESC3) lesions. The 12Z and PESCs were transfected with precursor miR‐29c‐3p (pre‐miR‐29c‐3p) to upregulate its expression, followed by functional assays. The effect of this miRNA on the expression of target genes was also investigated in the transfected cells, as well as in endometriosis patients. Building on our findings regarding the role of miR‐29c‐3p in endometrial cells, we used a miRNA database to predict potential targets of miR‐29c‐3p. We then selected targets that could explain our functional analysis results by reviewing the literature on their known roles in other cell types. These selected targets were further analyzed at both the mRNA and protein levels using qPCR and western blotting, respectively.

## 2 MATERIALS AND METHODS

### 2.1 Cell culture

12Z cells^33^ were cultured in Dulbecco's Modified Eagle's Medium (DMEM) (PAN Biotech, cat. no. P04‐01158, Aidenbach, Germany) with 10% FCS (Biochrom GmbH, cat. no. S0615, Berlin, Germany), 1% glutamine, and 1% penicillin–streptomycin (pen‐strep) (Sigma‐Aldrich, cat. no. P4333, Darmstadt, Germany) in a humidified atmosphere of 5% CO_2_ at 37°C. The primary endometriotic stromal cells were collected from three patients with endometriosis who underwent laparoscopy at Münster University Hospital between October 2012 and March 2014. The modified American Society for Reproductive Medicine (rASRM) classification was used to categorize the severity of endometriosis.^34^ Cells from the first patient (PESC1) were isolated from an endometriotic lesion in the rectovaginal septum of a 35‐year‐old patient, with multiple lesions, classified as rASRM Stage II. The second patient's cells (PESC2) were obtained from an endometriotic lesion on the uterine serosa of a 39‐year‐old woman, also classified as rASRM Stage II. Cells from the third patient (PESC3) were derived from a superficial peritoneal endometriotic lesion on the pelvic sidewall of a 19‐year‐old patient, classified as rASRM Stage III. To isolate the cells, a solution of 400 μL collagenase type CLS (50 mg/mL) (Sigma‐Aldrich), 100 μL DNase (10 mg/mL) (Sigma‐Aldrich), and 500 μL PBS (Sigma‐Aldrich) was added to the samples. The samples were kept for 1 h at 37°C, in 7.5% CO_2_, and 100% relative humidity in the CO_2_ incubator. Subsequently, 6 mL PBS with 10% FCS was added. The suspension was poured over another cell sieve, with the supernatant being collected in a 50 mL centrifuge tube. The stromal cells used for further experiments were in the flow‐through, while the epithelial cells could not pass through the sieve. The flow‐through was either frozen at −20°C or cultivated in DMEM containing 10% FCS, 1% glutamine, and 1% pen‐strep in a humidified atmosphere of 5% CO_2_ at 37°C, as explained before.^35^, ^36^ The local ethics commission (Ethikkommission der Ärztekammer Westfalen‐Lippe und der Medizinischen Fakultät der WWU; approval no. 1 IX Greb 1 from September 19, 2001, updated 2012) approved the study, and the subjects gave their written informed consent.

### 2.2 miRNA transfection

To ensure 80% confluency on the day of transfection, 2 × 10^5^ cells were seeded in 6‐well plates 24 h prior to transfection. Cells were transfected with Dharmafect reagent (Dharmacon, cat. no. T‐2001‐03, Lafayette, Colorado, USA) in OPTI‐MEM media (Gibco, cat. no. 31985‐070, Dreieich, Germany), using pre‐miR‐29c‐3p (10 nM) and pre‐miR precursor negative control #1 (10 nM) (Applied Biosystems, Darmstadt, Germany), according to the manufacturer's protocol. After 24 h, the transfection media were replaced by regular culture medium. Functional assays and gene expression analysis were performed 48–72 h after transfection.

### 2.3 Cell viability assay MTT

Cell proliferation was assessed by methyl thiazolyl diphenyl tetrazolium bromide (MTT) assay as illustrated before.^37^ In brief, 72 h after pre‐miR‐29c‐3p transfection, 20 000 12Z and 10 000 PESC cells were seeded in the first row of 96‐well plates and diluted 1:2 in every following row. The plates were incubated for 72 h, followed by a 4 h culture in the presence of MTT (Sigma‐Aldrich, cat. no. 298‐93‐1). After the 4 h incubation, a stop buffer was added, and the plates were kept at room temperature for 20 h, protected from light. The extinction was measured at 595 nm wavelength in a microplate reader (Molecular Devices, San Jose, California, USA). The experiment was performed six times in triplicate with the 12Z cells, three times with PESC1 cells, three times with PESC2, and four times with PESC3.

### 2.4 Cell cycle analysis

pre–miR‐29c‐3p‐transfected 12Z and PESC cells were trypsinized after 72 h of transfection along with their respective controls. A total of 50 μL of cell suspension was diluted with 950 μL DAPI (Sigma‐Aldrich, cat. no. D9564‐10MG). Following incubation at room temperature for 5 min, the cells were analyzed by flow cytometry using a CyFlow Space flow cytometer (Partec, Görlitz, Germany) equipped with a 375 nm UV laser for excitation. FlowMax software (Quantum Analytics, Munster, Germany) was used to calculate cell cycle phase distribution. The experiment was performed six times with 12Z cells, four times with PESC1 cells, three times with PESC2, and five times with PESC3.

### 2.5 Wound healing assay

Two hundred thousand 12Z cells/well of a six‐well plate were transfected with pre‐miR‐29c‐3p and pre‐miR precursor negative control for 24 h. After 48 h of transfection, a wound was created horizontally and vertically on the confluent layer using a pipette tip. Closing of the resulting cell‐free gap was documented with a Zeiss Axiophot camera (Zeiss, Gottingen, Germany) after 0, 6, 12, and 24 h, respectively, using Nomarski contrast light microscopy. NIH ImageJ software (NIH, Bethesda, MD, USA) was used for the quantification of cell‐free areas. The experiment was performed five times in duplicate.

### 2.6 Matrigel invasion assay

To measure cell invasiveness, 12Z cells (2.5 × 10^4^ cells/mL) were plated as duplicates into BD Matrigel™ Invasion Chambers (BD Biosciences, Franklin Lakes, NJ, USA) for 24 h of transfection. After 24 h, a chemotactic gradient was generated by applying serum‐free media to the upper chamber and DMEM with 10% FCS to the lower chamber. After 15–20 h, cells transmigrating through the filters were stained with Diff‐Quick (Medion Diagnostics AG, Düdingen, Switzerland) and cells in the upper chamber were removed using cotton swabs. Stained cells were photographed with a Zeiss Axiovert microscope (Zeiss) at 50 X magnification and counted in two central visual fields of each membrane. The experiment was performed three times in duplicate.

### 2.7 RNA isolation and reverse transcription

RNA was isolated from cells 72 h after transfection using the innuPREP RNA Mini Kit (Analytikjena, cat. no. 845‐KS‐2040250, Jena, Germany) following the manufacturer's protocol. The High‐Capacity cDNA Reverse Transcription Kit (Applied Biosystems, cat. no. 4368814) was used to convert mRNA into cDNA. miRNA was transcribed into cDNA using the TaqMan MicroRNA Reverse Transcription Kit (Applied Biosystems).

### 2.8 Quantitative TaqMan real‐time PCR analysis

To control for pre‐miR‐29c‐3p transfection efficiency, quantitative analysis of miR‐29c‐3p expression after 72 h transfection was assessed using a hsa‐miR‐29c‐3p TaqMan assay (Applied Biosystems, cat. no. 4427975) and TaqMan 2 × PCR Master mix (Applied Biosystems) in an ABI PRISM 7300 Real‐Time PCR System (Applied Biosystems). Real‐time PCR cycling conditions included one cycle of 2 min at 50°C, one cycle of 10 min at 95°C, and 40 cycles of 15 s at 95°C and 1 min at 60°C.

The possible primary targets of miR‐29c‐3p were searched using the mibase.org database^38^ and their expression in transfected cells was quantified using SYBR Select Master Mix (Applied Biosystems) with one cycle of 10 min at 95°C and 40 cycles of 15 s at 95°C and 1 min at 60°C on an ABI PRISM 7300 Real‐Time PCR System. Primer sequences are provided in Table 1. RNU6B (Applied Biosystems, cat. no: 4427975) and actin were used for miRNA and mRNA normalization, respectively. The fold change was calculated using the 2^–ΔCt^‐method.^39^

**TABLE 1:** SYBR green qPCR primers used in this study.

Gene name	Primer sequence
TCF7L1 forward primer	AAG GTG CCT GCC ACT TCC TC
TCF7L1 reverse primer	CCT GCC ACT CTG GGA TTG TG
TCF7L2 forward primer	AAA CCA GCT GCC GCT TTT ATG
TCF7L2 reverse primer	GCA ACA TCA ACA TGC CTA GGT TTT
PTEN forward primer	TGA GTT CCC TCA GCC GTT ACC T
PTEN reverse primer	GAG GTT TCC TCT GGT CCT GGT A
COL4A2 forward primer	CAG AAG TGG AGA CCT TTC TAG ACA TCA
COL4A2 reverse primer	GGG TTA AAT CTC AGG GAC AAC GTG
CDK6 forward primer	GCT GAC CAG CAG TAC GAA TG
CDK6 reverse primer	TGT TCG TGA CAC TGT GCA CA
Vimentin forward primer	TCA GCA TCA CGA TGA CCT TGA A
Vimentin reverse primer	CTG CAG AAA GGC ACT TGA AAG C
Actin forward primer	TCA AGA TCA TTG CTC CTC CTG AG
Actin reverse primer	ACA TCT GCT GGA AGG TGG ACA
ROBO1 forward primer	AGT GAG CCT CAG TTC ATC CAG C
ROBO1 reverse primer	GCT CCA ATA CCT GCT ATG AAG GC
p21 forward primer	AGG TGG ACC TGG AGA CTC TCAG
p21 reverse primer	TCC TCT TGG AGA AGA TCA GCC G
BCCIP forward primer	GAA GAG GAC GAG GTC ATT GAC G
BCCIP reverse primer	GCA GTG TTC ACA GGA GCC TTT AG
E‐Cadherin forward primer	CAA AGC CCA GAA TCC CCA AG
E‐Cadherin reverse primer	CAC ACC TGG AAT TGG GCA AA
N‐Cadherin forward primer	TTC TGA CAA CAG CTT TGC CTC TG
N‐Cadherin reverse primer	TTT ATT CAG AAC GCT GGG GTC A

### 2.9 Western blotting

RIPA buffer was used to generate protein lysates from cultured cells 72 h after transfection A total of 30–50 μg protein was separated by electrophoresis using 7.5% SDS polyacrylamide gels before electrotransfer to Protran Premium Nitrocellulose Blotting Membranes (GE Healthcare Life Sciences, Solingen, Germany). Nonspecific binding to the blot membranes was blocked by incubation with 2.5% nonfat dry milk for 1 h. The membranes were subsequently incubated with the primary antibodies (listed in Table 2) overnight at 4°C. After five washing steps with Tris‐buffered saline, the membranes were incubated with horseradish peroxidase‐labeled goat anti‐mouse IgG or goat anti‐rabbit IgG as a secondary antibody, as shown in Table 2. To generate the specific signal, the blotting membranes were incubated with an ECL reaction mix (SuperSignal® West Pico Chemiluminescent Substrate by Thermo Scientific, IL, USA), and signals were detected on X‐ray film. NIH ImageJ software (NIH) was used for densitometric analysis. The signal of proteins of interest was normalized to GAPDH or tubulin as housekeeping gene products after stripping with 0.2 mmol glycine buffer (pH 2.5), washing, and re‐incubation with primary antibody followed by the procedure described above. In the CDK6 and corresponding tubulin panel of Figure 6, the original image was mirrored (left–right) using Microsoft PowerPoint Software to show a loading order that is consistent with the other figures of the publication. This was necessary as the experimenter loaded the samples in the order miR‐29c‐3p (3×)—control (3×) in one experiment, in contrast to the other western blots.

**TABLE 2:** Antibodies used in western blot.

Antibody	Manufacturer	Cat. no.	Dilution
Rabbit anti‐human CDK6	Cell Signaling Technology, Danvers, MA, USA	13331	1:1000
Mouse anti‐human PTEN	Santa Cruz Biotechnology, Santa Cruz, CA, USA	7974	1:200
Rabbit anti‐human TCF4 (TCF7L2)	Cell Signaling Technology, Danvers, MA, USA	2569	1:1000
Rabbit anti‐human p21	Cell Signaling Technology, Danvers, MA, USA	2947	1:1000
Mouse anti‐human Tubulin	Sigma‐Aldrich, Steinheim, Germany	T5168‐.2ML	1:4000
Mouse anti‐human GAPDH	GeneTex, Radnor, PA, USA	627408	1:1000
Goat anti‐mouse	Calbiochem, Darmstadt, Germany	3872786	1:10 000
Goat anti‐rabbit	Calbiochem, Darmstadt, Germany	3816014	1:5000

### 2.10 EndometDB Turku Endometriosis database

To analyze miR‐29c‐3p target gene expression in clinical samples of endometriosis patients and control subjects, we employed EndometDB^32^ an interactive web‐based solution that collects the mRNA expression data from 115 patients and 53 controls, with over 24 000 genes and clinical features. EndometDB was utilized to analyze relative target mRNA expression in control endometrium compared to patient endometrium, ovarian endometrioma, peritoneal lesions, or deep endometriosis. Data were displayed using the box‐plot output option of the database. Details can be found in the original publication.^32^

### 2.11 Bioinformatic prediction of miR‐29c‐3p target genes

Predicted miR‐29c‐3p targets were identified using the bioinformatics platform microRNA database (https://www.mirbase.org/).^38^ Targets were investigated by qPCR and western blotting as described above.

### 2.12 Statistical analysis

**FIGURE 1:**
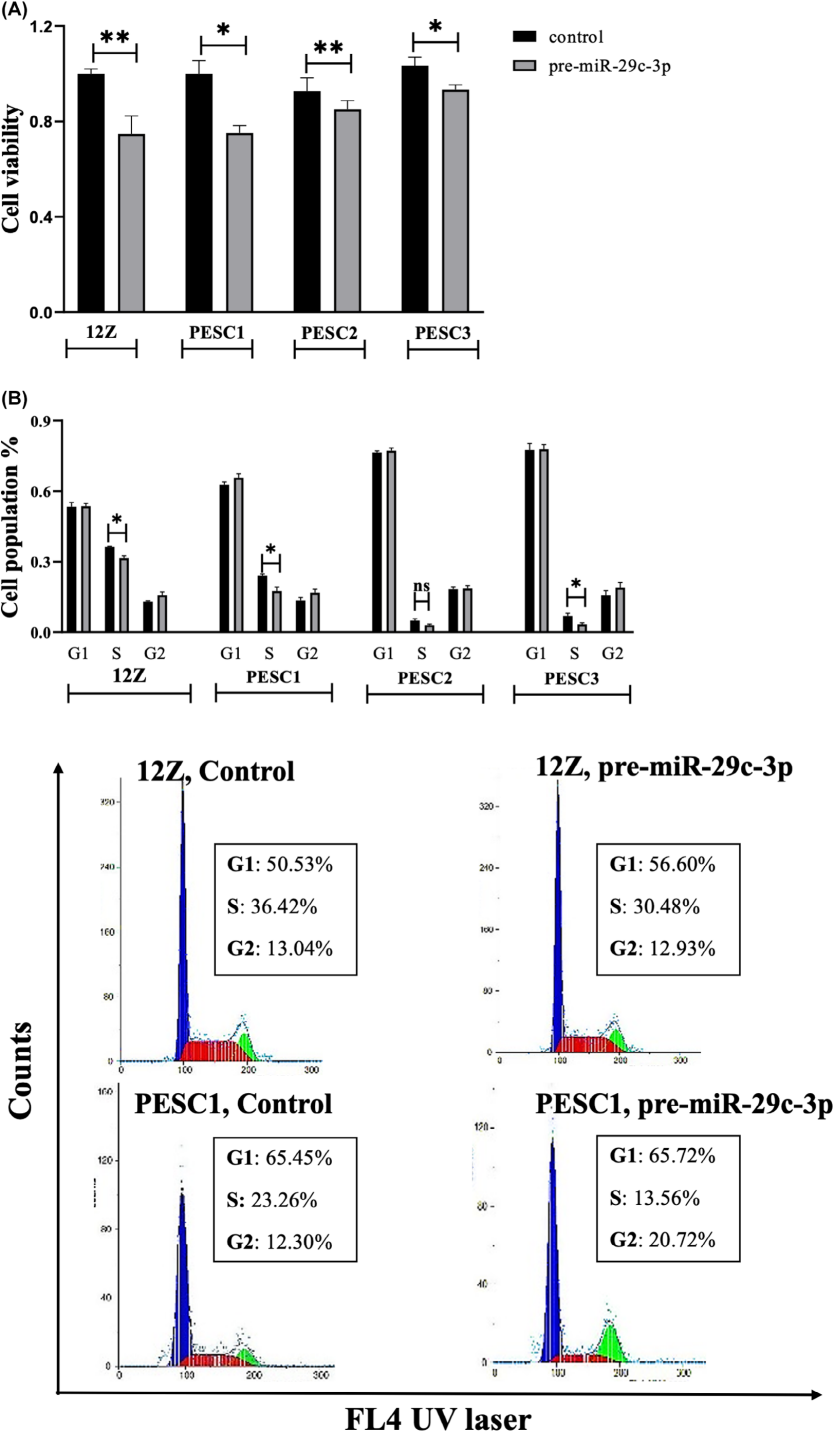
miR‐29c‐3p reduces endometriotic cell (12Z and PESC) viability and proliferation. (A) The MTT assay reveals a significant inhibition of 12Z, PESC1, PESC2, and PESC3 cell viability (*n* ≥ 3). (B) Upper panel: miR‐29c‐3p decreases the proportion of endometriotic cells in the S phase. Cell cycle phase composition was measured by means of DAPI staining and flow cytometry after 72 h of pre‐miR‐29c‐3p transfection (*n* = 6 for 12Z and *n* ≥ 3 for PESCs). Representative images of flow cytometric measurements are shown in the lower part of the figure. Data are presented as means ± SEM. Two‐tailed Student's *t*‐test, (*p < 0.05, **p < 0.01 compared to negative control‐transfected cells (control).

**FIGURE 2:**
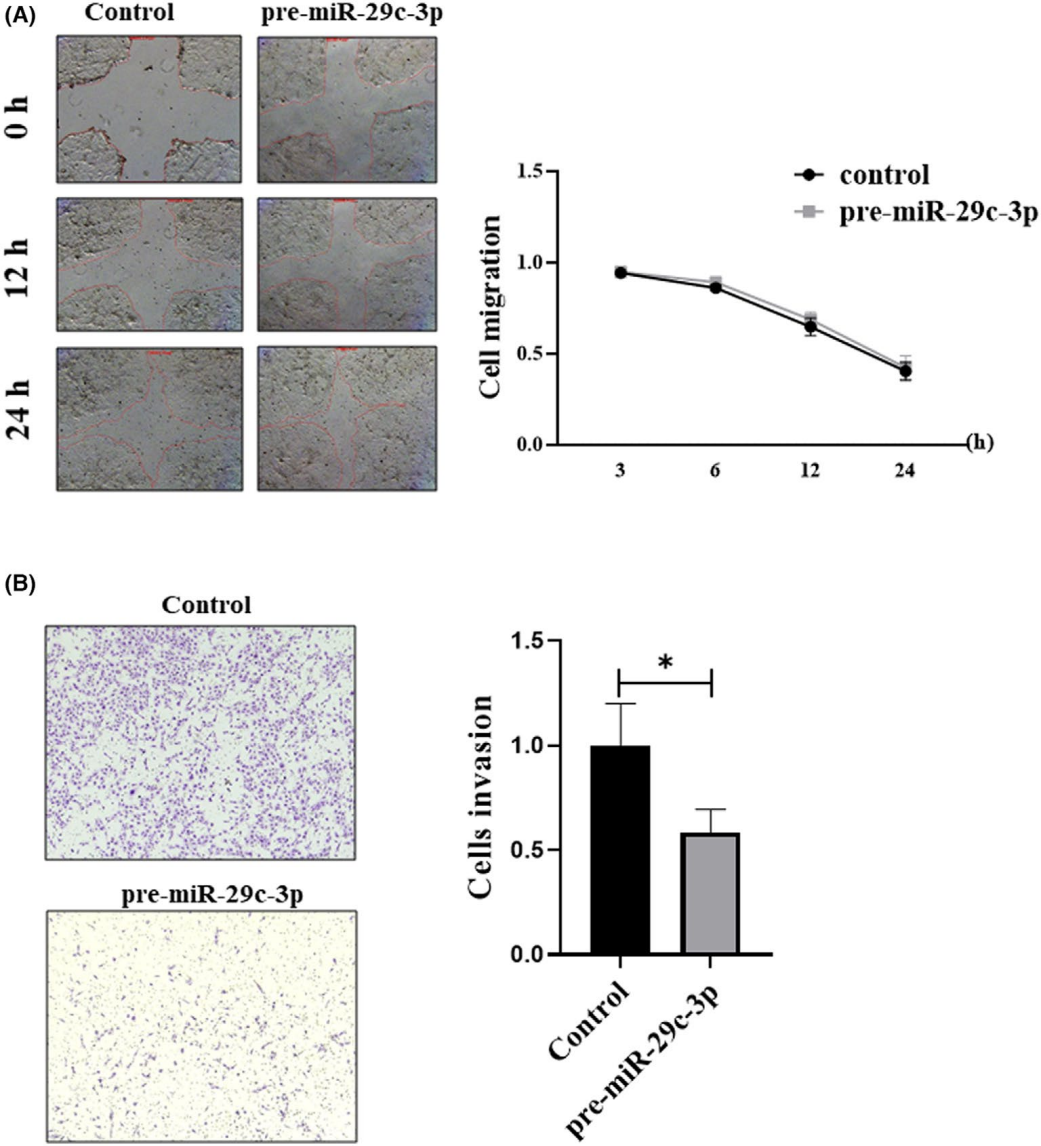
miR‐29c‐3p decreases 12Z endometriotic cell invasion, while it does not affect migration. (A) A nonsignificant change in cell migration was observed in a wound healing (scratch) assay. The left panel shows representative images of cell migration after 0, 12, and 24 h of the scratch (50×magnification) and the right panel shows the percentage of cell migration. Data are presented as means ± SEM. Two‐way ANOVA followed by Bonferroni multiple comparison test (*n* = 5). (B) 12Z cells transfected with pre‐miR‐29c‐3p had decreased invasion capacity compared to non‐transfected cells (control), as determined by transwell invasion assay (*n* = 3). The left panel presented representative images after 15–20 h of seeding the cells into the filters. The cells were seeded into the filters after 72 h of transfection. The invasive cells are stained purple, and the invasion filter pores can be seen as small gray dots. Photos were taken at 50× magnification. The right panel shows the percentage of cell invasion. Data are presented as means ± SEM. Student's *t*‐test. *p < 0.05 compared to negative control‐transfected cells (control).

**FIGURE 3:**
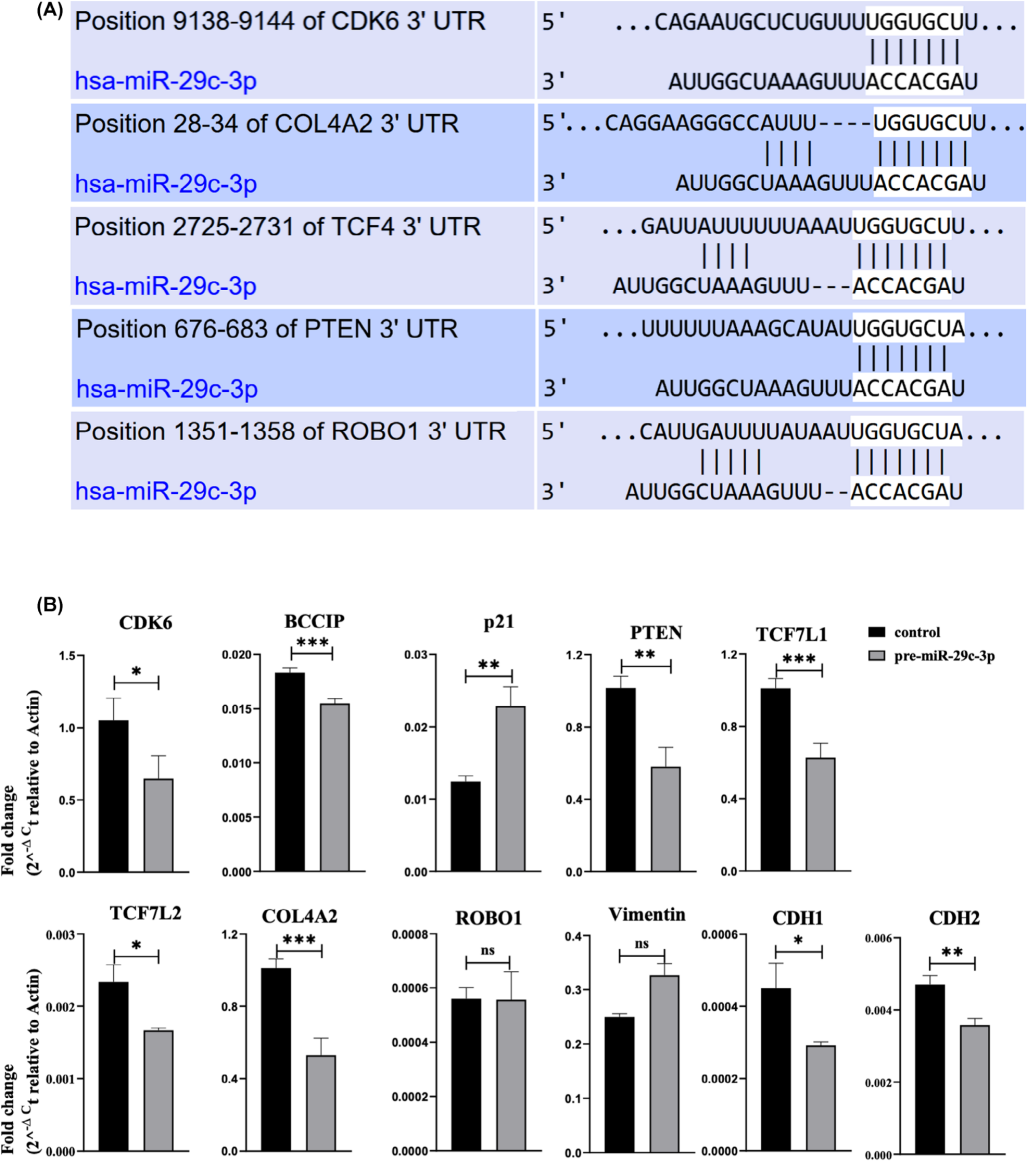
miR‐29c‐3p diminishes the expression of several mRNAs in 12Z epithelial endometriotic cells (A) Bioinformatic analysis by the Target Scan algorithm identifies CDK6, COL4A2, TCF4 (TCF7L2), PTEN, and ROBO1 as predicted targets of miR‐29c‐3p. Shown is an alignment of targets predicted binding sites of the miR‐29c‐3p seed sequence to the respective target mRNAs. Alignment data are taken from the targetscan.org. (B) The fold change expression of CDK6, BCCIP, p21, PTEN, TCF7L1, TCF7L2, COL4A2, ROBO1, and vimentin is compared to negative control‐transfected cells (control). Data are presented as means ± SEM (*n* = 3). Two tailed Student's *t*‐test (*p < 0.05, **p < 0.01, and ***p < 0.001).

**FIGURE 4:**
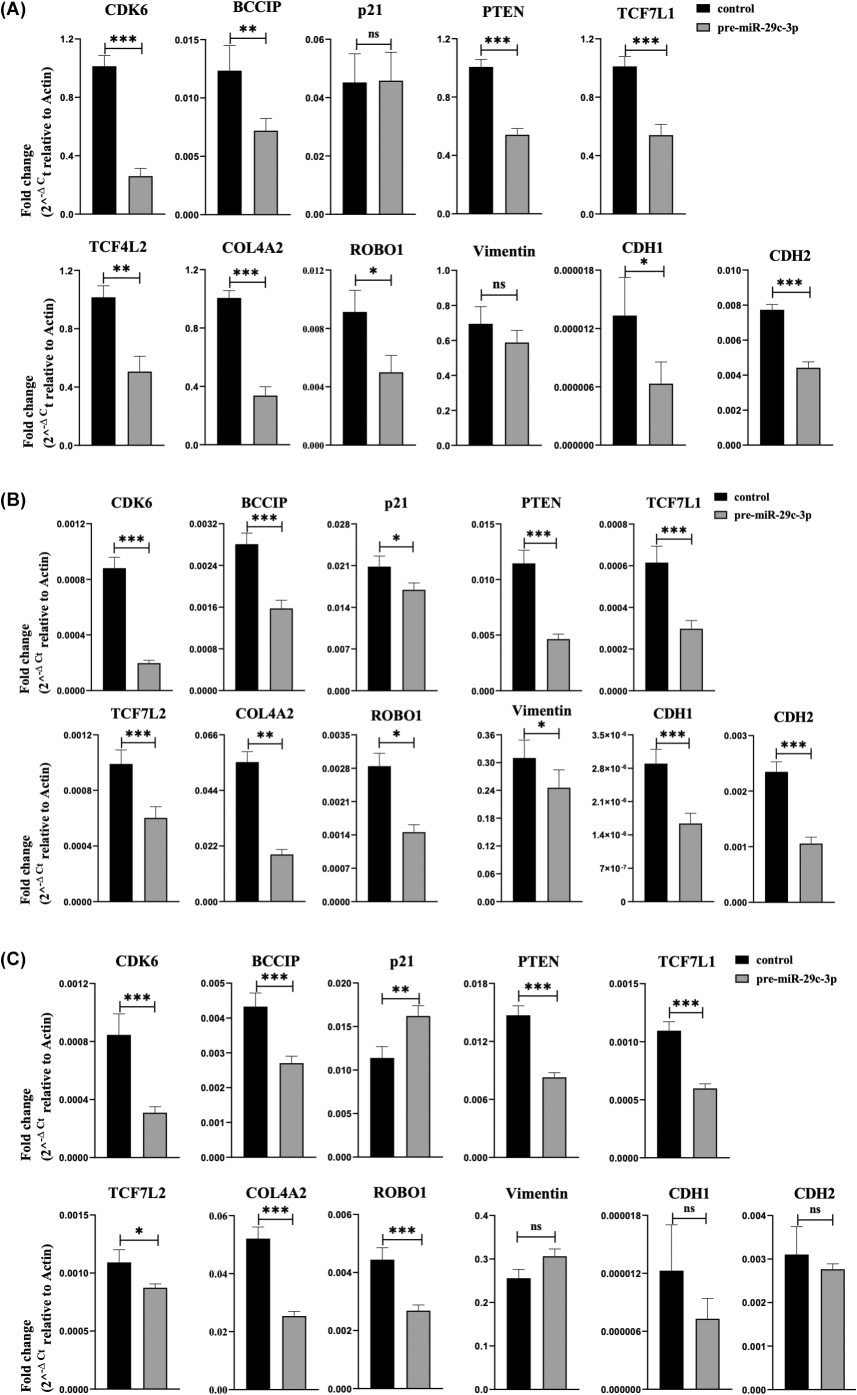
miR‐29c‐3p diminishes the expression of several mRNAs in primary endometriotic stromal cells. The fold change expression of *CDK6*, *BCCIP*, *p21*, *PTEN*, *TCF7L1*, *TCF7L2*, *COL4A2*, *ROBO1*, *vimentin*, *E‐Cadherin* (*CDH1*), and *N‐Cadherin* (*CDH2*) in pre‐miR‐29c‐3p‐transfected (A) primary endometriotic stromal cells isolated from patient 1 (PESC1), (B) primary endometriotic stromal cells isolated from patient 2 (PESC2), and (C) primary endometriotic stromal cells isolated from patient 3 (PESC3), compared to negative control‐transfected cells (control). Data are presented as means ± SEM (*n* = 3). Two‐tailed Student's *t*‐test (*p < 0.05, **p < 0.01, and ***p < 0.001).

**FIGURE 5:**
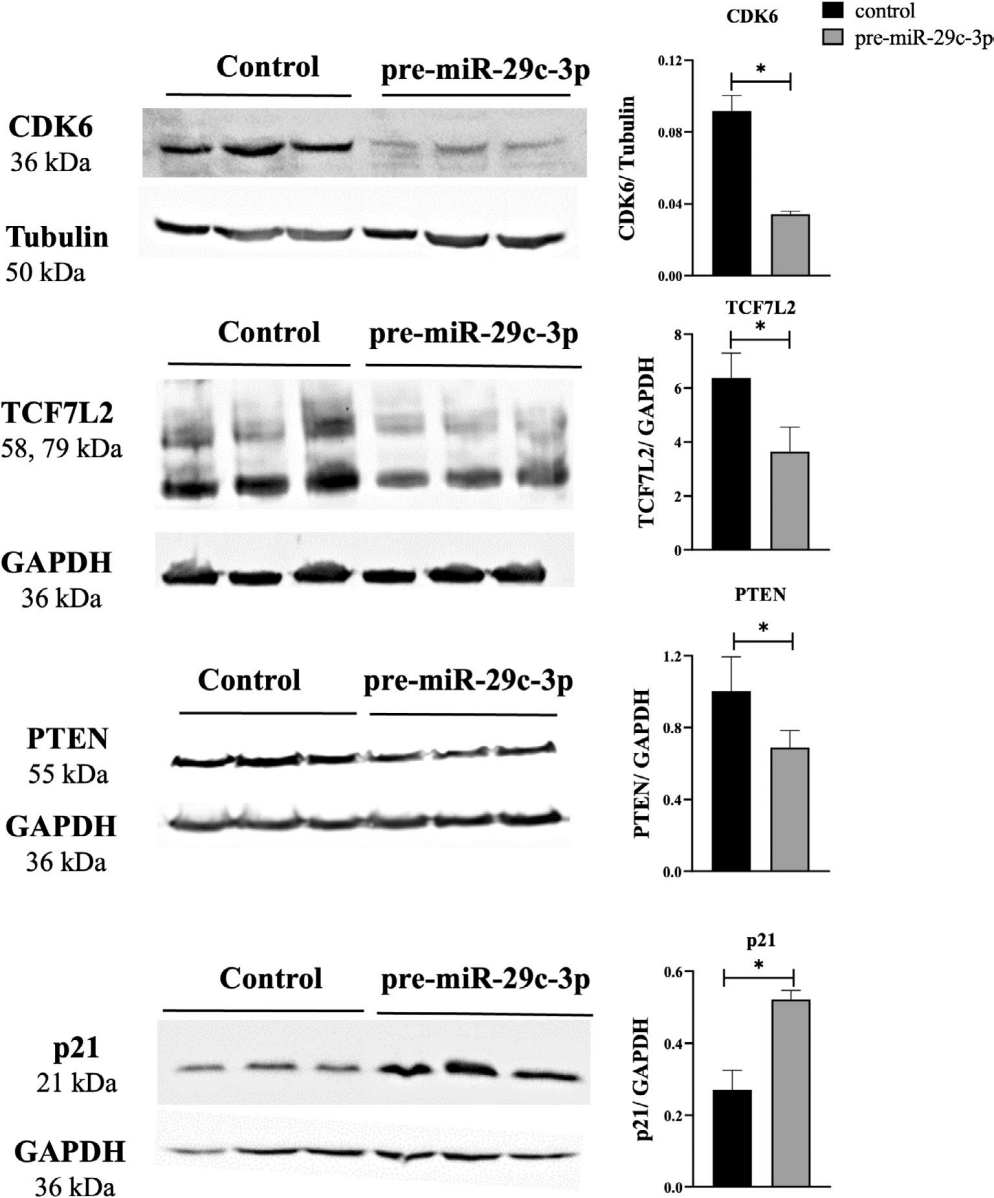
Impact of miR‐29c‐3p on protein expression in 12Z endometriotic cells. The protein expression of CDK6, TCF7L2, and PTEN is decreased in pre‐miR‐29c‐3p‐transfected 12Z cells after 72 h of transfection, and p21 protein is increased compared to negative control‐transfected cells (control). Representative bands are shown in the left panel, and the quantitative analysis via densitometric scanning is shown in the right panel (*n* = 3). Data represent the mean ± SEM. Two‐tailed Student's t‐test (*p < 0.05).

**FIGURE 6:**
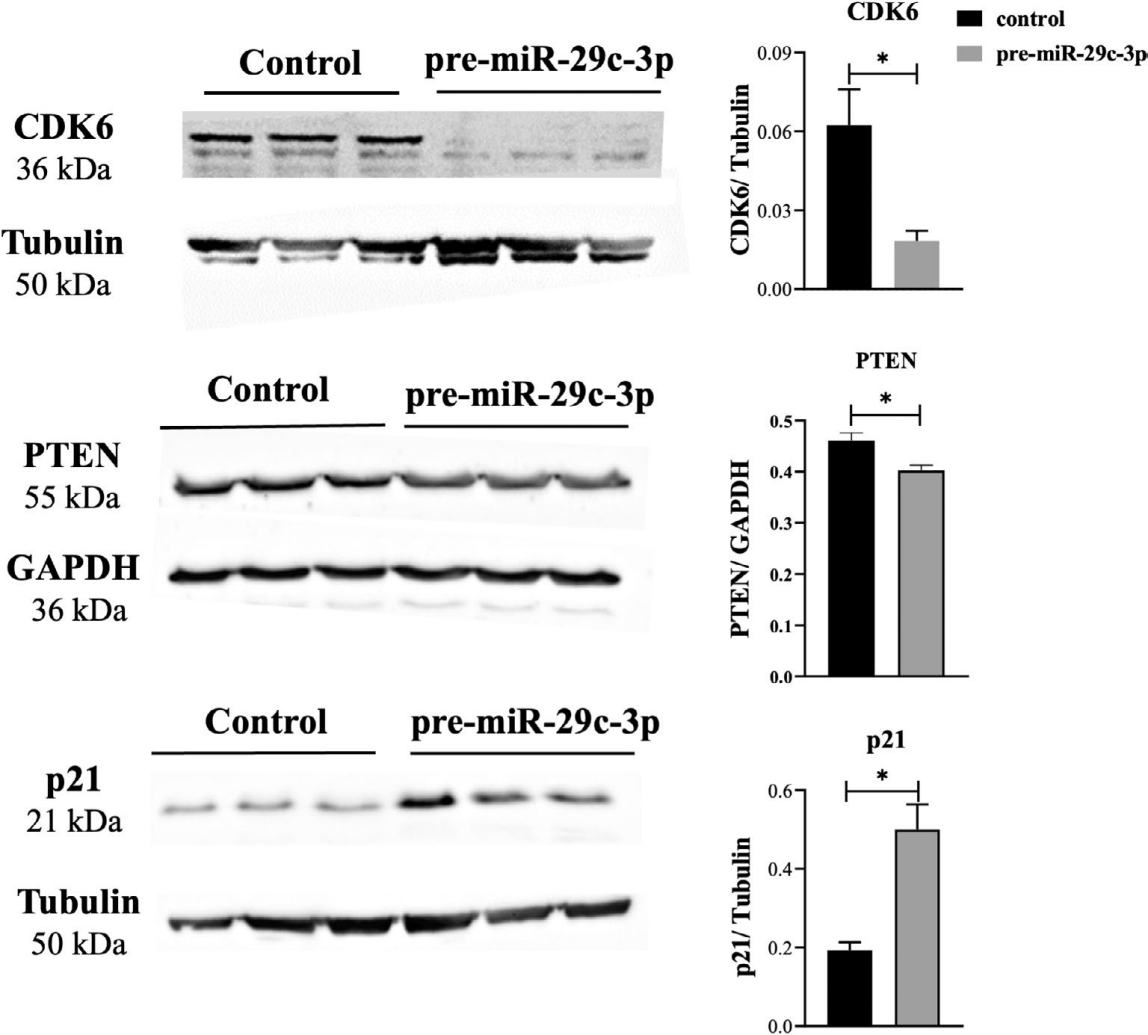
Impact of miR‐29c‐3p on protein expression in PESC1 primary endometriotic stromal cells. The protein expression of CDK6 and PTEN is decreased, and p21 is increased in pre‐miR‐29c‐3p‐transfected primary endometrial stromal cells isolated from patient 1. Representative bands are shown in the left panel, and the quantitative analysis via densitometric scanning is shown in the right panel (*n* = 3). In the CDK6 and corresponding tubulin panel, the original image was mirrored (left–right) to show a loading order that is consistent with the other figures of the publication, as described in methods. Data represent the mean ± SEM. Two‐tailed Student's t‐test (*p < 0.05). Data from primary endometrial stromal cells of patients 2 and 3 are shown in Figures S1 and S2, respectively.

**FIGURE 7:**
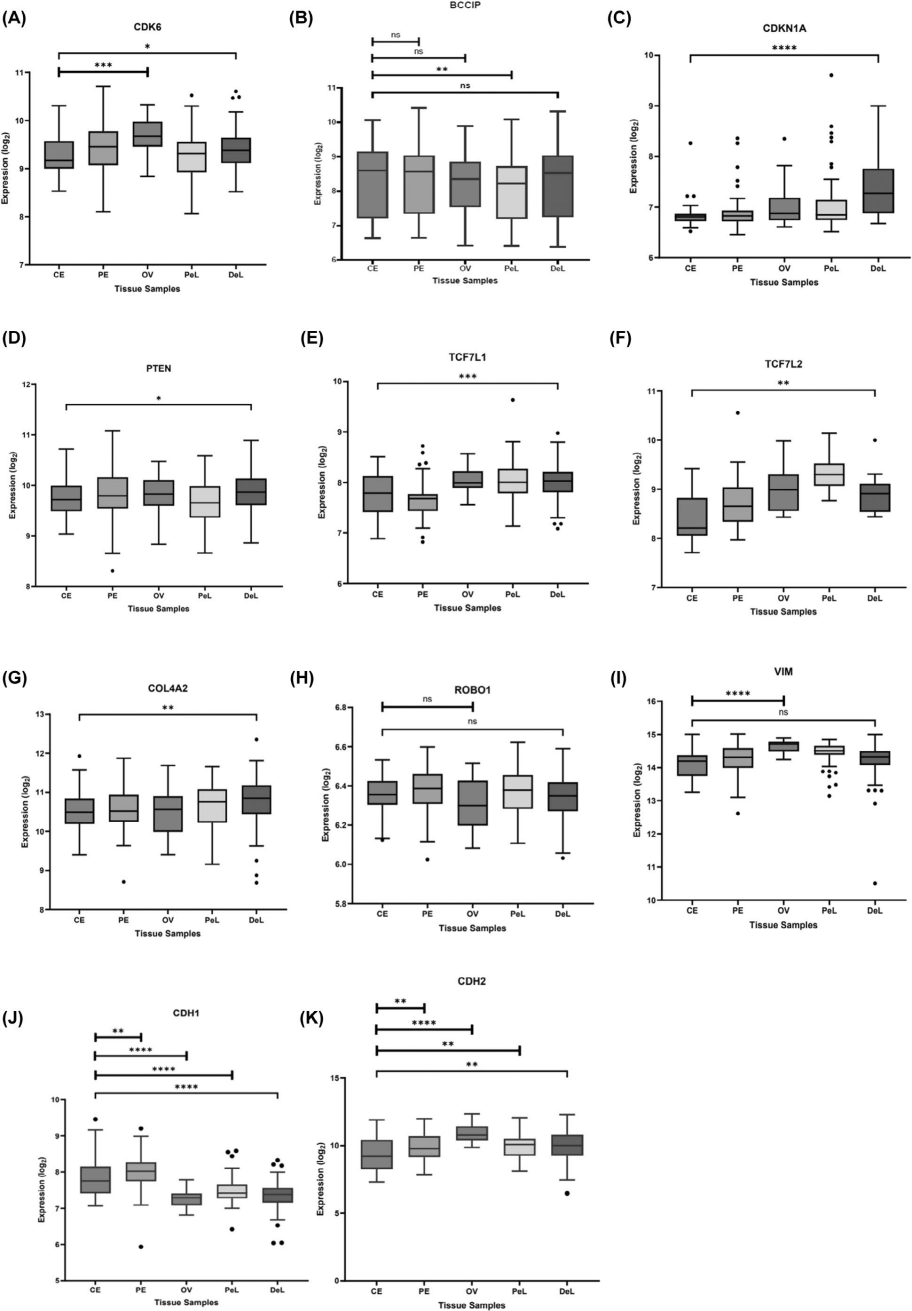
Gene expression analysis of miR‐29c‐3p targets in endometriotic lesions and control endometrium with the EndometDB Turku Endometriosis database. The web interface of the EndometDB^32^ was used to display mRNA‐based gene expression changes as Boxplots. The following sample groups were compared: CE = control endometrium (*n* = 40), PE = (endometriosis), patient endometrium (*n* = 89), OV = ovarian endometrioma (*n* = 27), PeL = peritoneal lesions (*n* = 71), and DeL = deep endometriosis lesions (*n* = 83). (*p < 0.05, **p < 0.01, ***p < 0.001, ****p < 0.0001, and ns = not significant). miR‐29c‐3p target gene names are displayed on top of the individual panels A–K.

Experiments were repeated independently at least three times. Values are expressed as mean ± standard error of the mean (SEM). Student's *t* test or two‐way ANOVA were used for statistical analysis as indicated in the figure legends (Figures 1, 2, 3, 4, 5, 6). For the gene expression data (EndometDB, Figure 7), multiple group comparisons statistics were performed using GraphPad Prism v.10.1. The distribution was tested using normality and lognormality tests, and the appropriate test selected based on distribution. Kruskal–Wallis test or one‐way ANOVA were used with the appropriate post hoc test (Dunnett's or Dunn's multiple comparison tests, respectively). *p < 0.05, **p < 0.01, ***p < 0.001, and ****p < 0.0001.

## 3 RESULTS

### 3.1 miR‐29c‐3p decreases endometriotic cell viability and cell cycle progression

To investigate a potential biological function of miR‐29c‐3p in our experimental system, we first used a transient transfection approach in the epithelial peritoneal endometriotic cell line 12Z and primary stromal endometriotic PESC1, PESC2, and PESC3 cells. The cells were transfected with negative control microRNA or pre–miR‐29c‐3p. Successful overexpression was confirmed by qRT‐PCR, normalizing miR‐29c‐3p expression to the small RNA RNU6B, resulting in a 10‐fold upregulation in 12Z cells and a 6–7fold upregulation in PESC cells after transfection (results not shown).

To investigate the cell viability, an MTT assay was carried out 72 h after transfection. A significant inhibition of cell viability in pre‐miR‐29c‐3p transfected 12Z, PESC1, PESC2, and PESC3 cells was observed (Figure 1A). To examine the cells with regard to their distribution to the different phases of the cell cycle, their DNA was first stained with DAPI. The assignment of the phases was based on a flow cytometric determination of the DNA content. In comparison to the control, the 12Z, PESC1, and PESC3 cells transfected with pre‐miR‐29c‐3p showed a lower proportion of cells in the S phase, while PESC2 did not show any significant change (Figure 1B).

### 3.2 miR‐29c‐3p reduces 12Z cell invasiveness but does not alter endometriotic cell migration

To examine the effect of miR‐29c‐3p on the migration ability of 12Z cells, the cells were first transfected, and then a wound healing assay was performed. The results revealed that there was no significant difference in the migration ability between transfected and non‐transfected cells (Figure 2A). A matrigel invasion assay was utilized to measure the effect of miR‐29c‐3p on the invasion capacity of 12Z. Light microscopy‐based quantification of the cells migrating through the membrane showed that the invasiveness of the 12Z endometriosis cells transfected with pre‐miR29c‐3p was reduced to 56.5% compared to their respective control (Figure 2B).

### 3.3 miR‐29c‐3p inhibits the expression of several predicted targets

The results of the functional analysis of miR‐29c‐3p on endometrial cells showed that miR‐29c‐3p reduced the cells' invasion, viability, and proliferation. Therefore, we applied an in‐silico approach to search for the predicted target genes of this miRNA, which are involved in generating the previously mentioned effects. Using the Target Scan algorithm included in miRbase,^38^, ^40^ several targets were identified which displayed miR‐29c‐3p targeting sites in their 3′UTRs (Figure 3A). Adding additional targets based on literature data^41^ and filtering the data for their potential relevance for the observed miR‐29c‐3p‐induced phenotypes of this study, the following potential targets were used for further analysis by qPCR: *CDK6*, *BCCIP*, *TCF7L1*, *TCF7L2*, *PTEN*, *COL4A2*, *ROBO1*, *vimentin*, *E‐Cadherin*, and *N‐Cadherin*. RNA and protein lysates were collected from miR‐29c‐3p transfected 12Z and PESCs cells as well as their respective negative controls after 72 h of transfection. qPCR analysis revealed that in 12Z cells, *CDK6*, *BCCIP*, *PTEN*, *TCF7L1*, *TCF7L2*, *COL4A2*, *E‐Cadherin*, and *N‐Cadherin* were significantly downregulated in pre‐miR‐29c‐3p‐transfected cells; however, *p21* expression was significantly upregulated, and *vimentin* and *ROBO1* expression did not significantly change in pre‐miR‐29c‐3p transfected cells (Figure 3B). In PESC1 cells, *CDK6*, *BCCIP*, *PTEN*, *TCF7L1*, *TCF7L2*, *COL4A2*, *ROBO1*, *E‐Cadherin*, and *N‐Cadherin* were significantly downregulated in pre‐miR‐29c‐3p‐transfected cells with no significant change in *vimentin* and *p21* expression (Figure 4A). In PESC2 cells, *CDK6*, *BCCIP*, *p21*, *TCF7L1*, *TCF7L2*, *PTEN*, *COL4A2*, *ROBO1*, *vimentin*, *E‐Cadherin*, and *N‐Cadherin* were significantly downregulated in pre‐miR‐29c‐3p‐transfected cells (Figure 4B). In PESC3 cells, *CDK6*, *BCCIP*, *PTEN*, *TCF7L1*, *TCF7L2*, *COL4A2*, and *ROBO1* were significantly downregulated in pre‐miR‐29c‐3p‐transfected cells with no significant change in *vimentin*, *E‐Cadherin*, and *N‐Cadherin*. However, *p21* expression was significantly upregulated (Figure 4C).

The expression of the potential targets was further examined at the protein level by western blotting. In 12Z cells, CDK6, TCF7L2, and PTEN were significantly downregulated. Moreover, cyclin‐dependent kinase inhibitor p21 was significantly upregulated in pre‐miR‐29c‐3p transfected cells compared to the respective control (Figure 5). In PESC1 cells, the expression of CDK6 and PTEN was significantly downregulated, and p21 expression was significantly upregulated in pre‐miR‐29c‐3p‐transfected cells (Figure 6). Likewise, in PESC2 cells, the expression of CDK6 and PTEN was significantly downregulated, and p21 expression was significantly upregulated in pre‐miR‐29c‐3p‐transfected cells (Figure S1). In PESC3 cells, the expression of CDK6 and PTEN was significantly downregulated, and p21 expression was significantly upregulated in pre‐miR‐29c‐3p‐transfected cells (Figure S2).

### 3.4 The expression of miR‐29c‐3p targets is dysregulated in endometriosis

Finally, we investigated the expression of miR‐29c‐3p targets in deep endometriosis (bladder, intestine, rectovaginal, and sacrouterine ligament), peritoneal lesions, ovarian endometriosis, and eutopic endometrium of endometriosis patients in comparison to healthy endometrium utilizing the Turku EndometDB database, which incorporates gene expression data from 115 patients and 53 controls.^32^ In accordance with our functional observation of reduced invasiveness of miR‐29c‐3p‐transfected endometriotic cells, a significant upregulation of its target genes *CDK6*, *p21* (*CDKN1*), *PTEN*, *TCF7L1*, *TCF7L2*, *COL4A2*, and *N‐Cadherin* was detected in lesions of deep endometriosis compared to control endometrium. In addition, a significant upregulation of *CDK6*, *vimentin*, and *N‐Cadherin* expression was noted in ovarian endometrioma compared to control endometrium. In contrast, the expression of *BCCIP* and *E‐Cadherin* was significantly downregulated in peritoneal lesions, and we did not observe any significant changes in *ROBO1* expression between control and endometriotic tissue (Figure 7).

## 4 DISCUSSION

In the current study, we employed 12Z and PESCs as models of peritoneal epithelial and stromal endometrial cells, respectively, to investigate the roles of miR‐29c‐3p in endometrial cell function. The cells were transfected with pre‐miR‐29c‐3p to upregulate the expression of miR‐29c‐3p, which was confirmed by qPCR. The cells were then utilized for functional analysis in which the transfected cells had a significant reduction in their proliferation, invasion, and proportion in the S phase of the cell cycle. Moreover, a significant downregulation of several potential targets, including *CDK6*, *BCCIP*, *PTEN*, *TCF7L1*, *TCF7L2*, *ROBO1*, *COL4A2*, *E‐Cadherin*, and *N‐Cadherin*, was observed in the transfected cells and endometriosis patients.

miR‐29c‐3p has been observed in various cancers to be strongly associated with proliferation, invasion, and migration. For instance, reduced migration, invasion, colony formation, and proliferation with overexpression of miR‐29c‐3p in mesothelioma were reported.^42^ In endometrial cancer type 1, miR‐29c is downregulated and its overexpression in endometrial cancer cell lines resulted in reduced proliferation and increased apoptosis.^43^ Several studies have reported a dysregulated expression of miR‐29c in endometriosis. In some studies, it was shown to be upregulated^16^, ^20^, ^21^, ^22^, ^24^ and others reported its downregulated expression.^23^, ^25^ This could be attributed to the discrepancies in the target group and target samples under investigation. For instance, miR‐29c‐3p expression in eutopic and peritoneal ectopic endometrial tissue from seven patients with endometriosis was shown to be upregulated in the study by Ohlsson Teague et al.^16^ Braza‐Boïls et al. investigated the expression of miR‐29c‐3p in peritoneal lesions compared to healthy endometrium and reported a significant upregulation of the miRNA.^22^ However, the expression of miR‐29c‐3p was downregulated in the peritoneal fluid of endometriosis patients compared to the endometrial fluid obtained from healthy fertile patients, as reported by Marí‐Alexandre et al.^25^ Nevertheless, Long et al. reported a strong association between miR‐29c‐3p and reduced endometrial cell proliferation, viability, and invasion.^23^ In their research, they used the CRL‐7566 endometriosis cell line as a model of ovarian endometriosis to study the functions of miR‐29c‐3p. Our study provides more information on miR‐29c‐3p functions in the peritoneal epithelial endometriosis model and primary stromal endometrial cells.

To shed light on the mechanisms behind the observed decrease in cell proportion in S phase and viability, we delved into the potential miR‐29c‐3p targets such as CDK6, BCCIP, and PTEN. CDK6 belongs to the group of cyclin‐dependent kinases (CDKs) which are activated after binding to the corresponding cyclins. The cyclin‐CDK complexes enable the cells transition from G1 to S phase.^44^ A downregulated expression of miR‐29c was observed in gastric carcinomas. Reduced invasion and migration due to CDK6 suppression and miR‐29c upregulation was shown.^45^ We have seen that CDK6 was significantly downregulated in 12Z, PESC1, PESC2, and PESC3 after miR‐29c‐3p upregulation at both mRNA and protein levels. We have found a significant upregulation of CDK6 in deep endometriosis lesions and ovarian endometriosis lesions compared to healthy endometrium tissue. This agrees with previous studies in which CDK6 was reported to be significantly upregulated in the eutopic endometrium of endometriosis patients compared to healthy endometrium.^46^ Moreover, CDK6 was shown to be significantly upregulated in eutopic and ectopic endometriosis patients compared to their healthy control.^47^

Given the inhibitory effect of p21 on CDK6 activity, as reported in previous studies,^48^ we selected p21 for further investigation. Our results showed a significant upregulation of p21 expression in pre‐miR‐29c‐3p transfected 12Z, PESC1, PESC2, and PESC3 at the protein level. Likewise, a significant upregulation of p21 expression was found in deep endometriosis compared to healthy endometrium. In line with our findings, PESCs exhibited increased proliferation and invasion after p21 inhibition, which was accompanied by an upregulation of miR‐202 expression. However, p21 expression in ectopic endometriosis was decreased.^49^ Nevertheless, other studies showed an increased expression of p21 in ovarian endometriosis compared to peritoneal and colorectal endometriosis.^50^ Following in silico analysis of p21 inhibitors, we identified BRCA2 and CDKN1A interacting protein (BCCIP) as one of the p21 modulators,^51^, ^52^ which is a predicted primary target of miR‐29c‐3p. Therefore, we examined the level of BCCIP expression, which resulted in a significant decrease in its expression in pre‐miR‐29c‐3p‐transfected 12Z, PESC1, PESC2, and PESC3. We additionally found a downregulation of BCCIP expression in peritoneal lesions of endometriosis patients compared to healthy endometrium. This suggests that the targeting of BCCIP by miR‐29c‐3p may have resulted in the increased expression of p21, which could ultimately inhibit CDK6, leading to the inhibition of proliferation.

Phosphatase and tensin homolog deleted on chromosome 10 (PTEN) is a tumor suppressor that inhibits cell growth and migration.^53^, ^54^ We observed a significant reduction of PTEN in the transfected 12Z and PESCs at both mRNA and protein levels. Moreover, we have found an elevated expression of PTEN in deep endometriosis, which agrees with a previously published study that illustrated an increased PTEN expression in peritoneal lesions compared to eutopic endometrium.^55^ A reduced PTEN expression in endometriosis patients compared to healthy endometrium was additionally shown.^56^ Altogether, we have observed a reduced proliferation and invasion in pre‐miR‐29c‐3p‐transfected cells with a significant decrease in PTEN expression. This clearly contradicts current findings on the role of PTEN in both processes and might suggest that the association between PTEN and miR‐29c‐3p could affect other endometrial cellular processes such as apoptosis and angiogenesis. Evidence from the literature showed that miR‐29c‐3p regulates apoptosis and angiogenesis.^57^, ^58^, ^59^ Further studies are needed to elucidate this relationship in endometrial cells.

Epithelial–mesenchymal transition (EMT) is a reversible transition from cells with epithelial properties to cells with mesenchymal properties. EMT is an essential process for the proper function of the human endometrium, including endometrium receptivity.^60^ Dysregulated EMT is involved in endometriosis etiology.^61^, ^62^ Moreover, miR‐29c‐3p was reported to inhibit EMT in human cervical cancer cells.^63^ Therefore, a group of potential primary targets of miR‐29c‐3p, which are involved in EMT, has been selected for further analysis, such as TCF7L1, TCF7L2, COL4A2, and ROBO1.

The TCF/LEF family, comprising T‐cell factor and lymphoid enhancer factor genes, encodes transcription factors that play a critical role in the Wnt signaling pathway, particularly during embryonic development.^64^ Humans have four genes encoding TCF/LEF proteins, including TCF7, LEF1, TCF7L1 (also called TCF3), and TCF7L2 (also called TCF4).^65^ Our in vitro experiments presented a significant downregulation of *TCF7L1* and *TCF7L2* in pre‐miR‐29c‐3p‐transfected 12Z, PESC1, PESC2, and PESC3. In endometrial carcinosarcomas, TCF7L2 is upregulated in the mesenchymal compared to the epithelial component^66^ confirming its role in EMT. It was also reported that inhibiting TCF resulted in the decrease of invasion and migration in endometrial cells obtained from endometriosis patients.^67^

Collagen, Type IV, Alpha 2 (COL4A2) is a component of the extracellular matrix, forming a heterotrimer together with COL4A1 that is essential for the stability of the vascular basement membrane. It provides structural support and participates in cell–matrix communication via interactions with cell surface receptors and growth factors.^68^, ^69^ In the present study, *COL4A2* expression was significantly downregulated in pre‐miR‐29c‐3p‐transfected 12Z, PESC1, PESC2, and PESC3 cells. We have found upregulated *COL4A2* expression in deep endometriosis compared to healthy endometrium. Consistent with our findings, COL4A2 is upregulated in ovarian endometriosis compared to healthy ovarian tissues.^70^ COL4A2 is directly and significantly related to the EMT process in glioma^71^ and laryngeal squamous cell carcinoma.^72^

Slit glycoproteins are released by midline glia and function by interacting with single‐pass transmembrane proteins of the Roundabout family (ROBO1‐4).^73^ The Slit‐Robo signaling pathway plays crucial roles in directing neuronal connections and the migration of diverse cell types.^74^ Our data showed a downregulated expression of ROBO1 in pre‐miR‐29c‐3p transfected PESC1, PESC2, and PESC3 with no significant difference in 12Z. No significant difference in ROBO1 expression in endometriosis patients was also seen. Nevertheless, Slit‐Robo1 was reported to be upregulated in the cases of recurrence ovarian endometriosis compared to non‐recurrence and healthy controls.^75^

Considering vimentin, E‐Cadherin, and N‐Cadherin essential roles in EMT,^76^, ^77^, ^78^ they were additionally investigated, although they are not a potential primary target for miR‐29c‐3p. In the present study, an insignificant change in vimentin expression was observed in pre‐miR‐29c‐3p‐transfected 12Z, PESC1, and PESC3, while it was significantly downregulated in PESC2. The insignificant change in vimentin expression was also seen in deep endometriosis; however, we saw upregulated vimentin expression in ovarian endometriosis compared to healthy endometrium. Elevated vimentin expression in ovarian endometriosis was also reported by previous studies.^79^, ^80^ Moreover, the expressions of *E‐Cadherin* and *N‐Cadherin* were significantly downregulated in 12Z, PESC1, and PESC2, with no significant change observed in their expression in PESC3. E‐Cadherin plays a key role in maintaining the epithelial state, and its downregulation, similar to what is observed in tumor cells, has been suggested as a molecular mechanism that enables endometrial cells to detach from their original location in cases of endometriosis. This detachment facilitates the adhesion and invasion of these cells at pelvic implantation sites, leading to the formation of endometriotic lesions.^81^ However, research on E‐Cadherin expression in endometriosis has yielded conflicting results. While some studies report a reduction in E‐Cadherin levels,^82^ others show no significant downregulation^83^ or even an increase, particularly in cases of deep infiltrating endometriosis and black peritoneal lesions.^84^ This variability could explain the reduced E‐Cadherin expression observed in our study, despite a decrease in invasion. This prompted us to investigate N‐cadherin, a mesenchymal marker that is crucial during cell migration.^85^ Our findings revealed a significant reduction in N‐Cadherin expression, which may explain the decreased invasion observed in 12Z cells, promoting a shift toward reduced EMT. These results are consistent with the findings of other researchers, who also observed reduced invasion and EMT with the downregulated expression of N‐Cadherin.^86^, ^87^

Collectively, these data suggest that downregulating TCF7L1, TCF7L2, COL4A2, and N‐Cadherin via upregulating the expression of miR‐29c‐3p in endometrial cells could explain the reduction in cell invasion we obtained in pre‐miR‐29c‐3p‐transfected cells.

**FIGURE 8:**
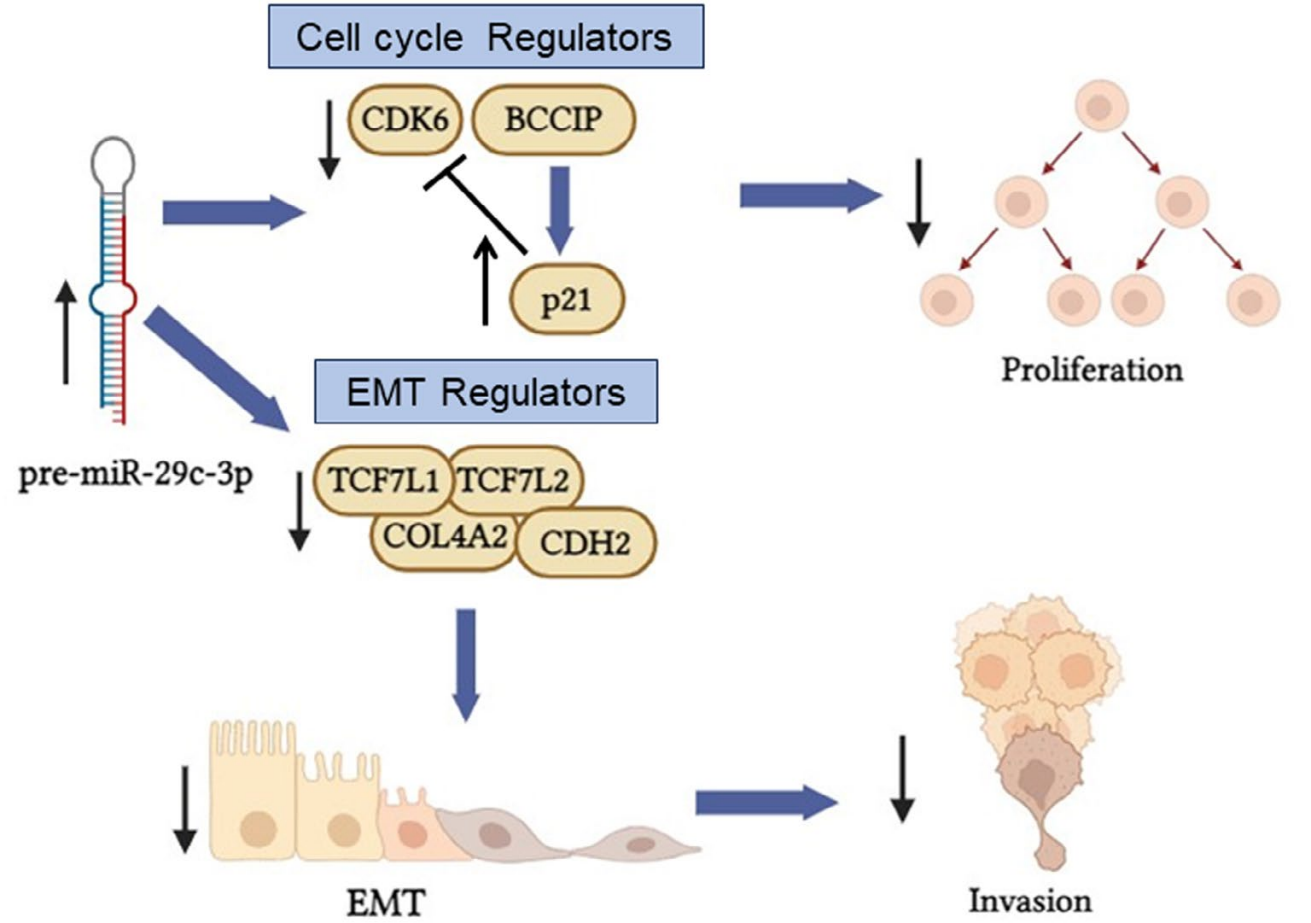
Model of miR‐29c‐3p's impact on endometriotic cell proliferation and invasion. Upregulation of miR‐29c‐3p leads to the downregulation of cell cycle regulators, including CDK6 and BCCIP (an inhibitor of p21), resulting in the upregulation of p21. This, in turn, inhibits CDK6, significantly reducing cell proliferation. Additionally, miR‐29c‐3p downregulates epithelial–mesenchymal transition (EMT) regulators such as TCF7L1, TCF7L2, COL4A2, and N‐Cadherin (CDH2), potentially reducing EMT and thereby inhibiting invasion. Blue arrows indicate the mechanistic effects of miR‐29c‐3p. ↑ corresponds to upregulation/increase, and ↓ corresponds to downregulation/decrease. The figure was created using BioRender (www.biorender.com).

Altogether, the present study has demonstrated that miR‐29c‐3p effectively inhibits endometrial cell proliferation and invasion by downregulating the expression of CDK6, BCCIP, TCF7L1, TCF7L2, COL4A2, and N‐Cadherin (Figure 8). Notably, these molecules were also found to be dysregulated in endometriosis patients, suggesting a potential link between their dysregulation and the development of endometriosis. Furthermore, the observed reduction in EMT induced by miR‐29c‐3p highlights its potential role in modulating endometrial function. Given these findings, miR‐29c‐3p and the identified downstream targets warrant further investigation as promising therapeutic targets for endometriosis.

## CONFLICT OF INTEREST STATEMENT

The authors declare no conflict of interest.

## ETHICS STATEMENT

The local ethics commission (Ethikkommission der Ärztekammer Westfalen‐Lippe und der Medizinischen Fakultät der WWU; approval no. 1 IX Greb 1 from September 19, 2001, updated 2012) approved the study, and the subjects gave their written informed consent.

## HUMAN RIGHTS STATEMENTS AND INFORMED CONSENT

All procedures followed were in accordance with the ethical standards of the committee responsible for human experimentation (institutional and national) and with the Helsinki Declaration of 1964 and its later amendments.

## Supporting information


Figure S1.

Figure S2.

## Data Availability

To facilitate comprehensive gene expression analysis, publicly available patient data were retrieved from the ENDOMET Turku Endometriosis Database (https://endometdb.utu.fi/, accessed 20/07/2023). This comprehensive resource, developed by,^8^ provides a wealth of information on endometriosis‐related gene expression patterns. Expression data are available from the Gene expression omnibus (GEO) GSE141549.
