# Supplementary figures and images for: Combined targeting of TCF7L1/2, PTEN, CDK6, and BCCIP by microRNA miR‐29c‐3p is associated with reduced invasion and proliferation of endometriotic cells
Source: Reprod Med Biol. 2025 Mar 25;24(1):e12645. doi: 10.1002/rmb2.12645 (PMC11933757; doi:10.1002/rmb2.12645)

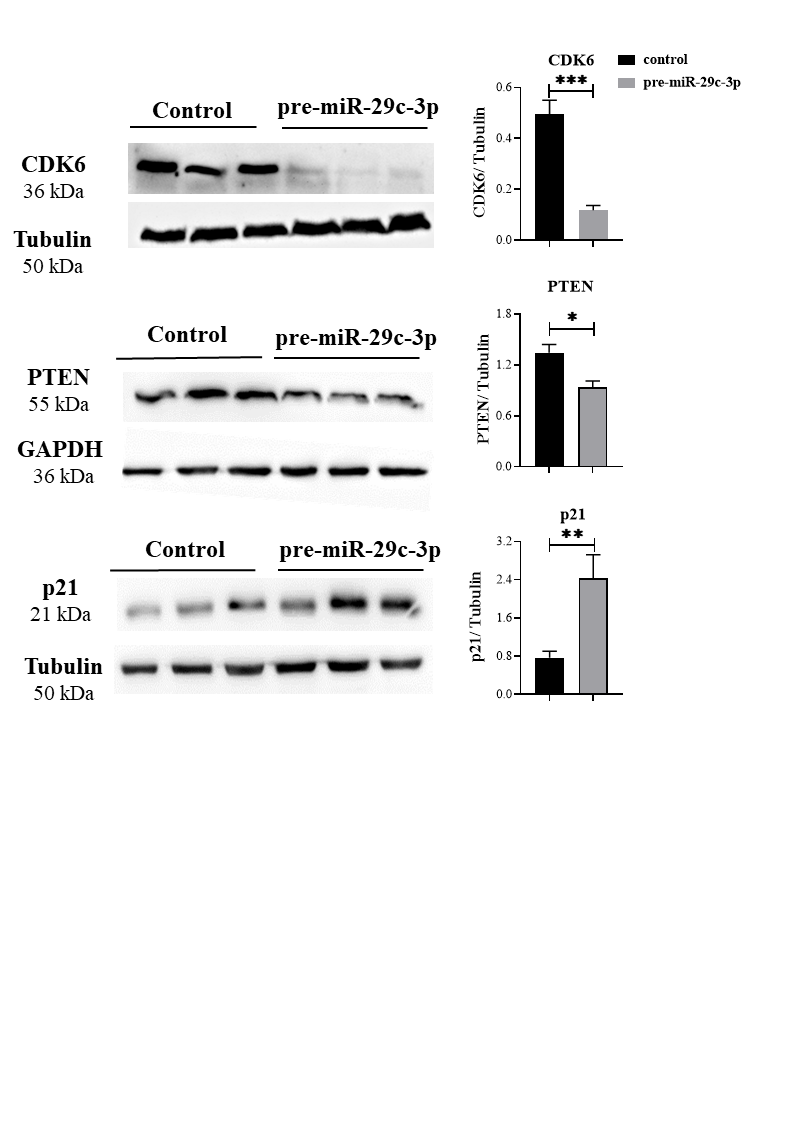

Supplement: Supplementary file 1 — Figure S1. [file RMB2-24-e12645-s002.tif]

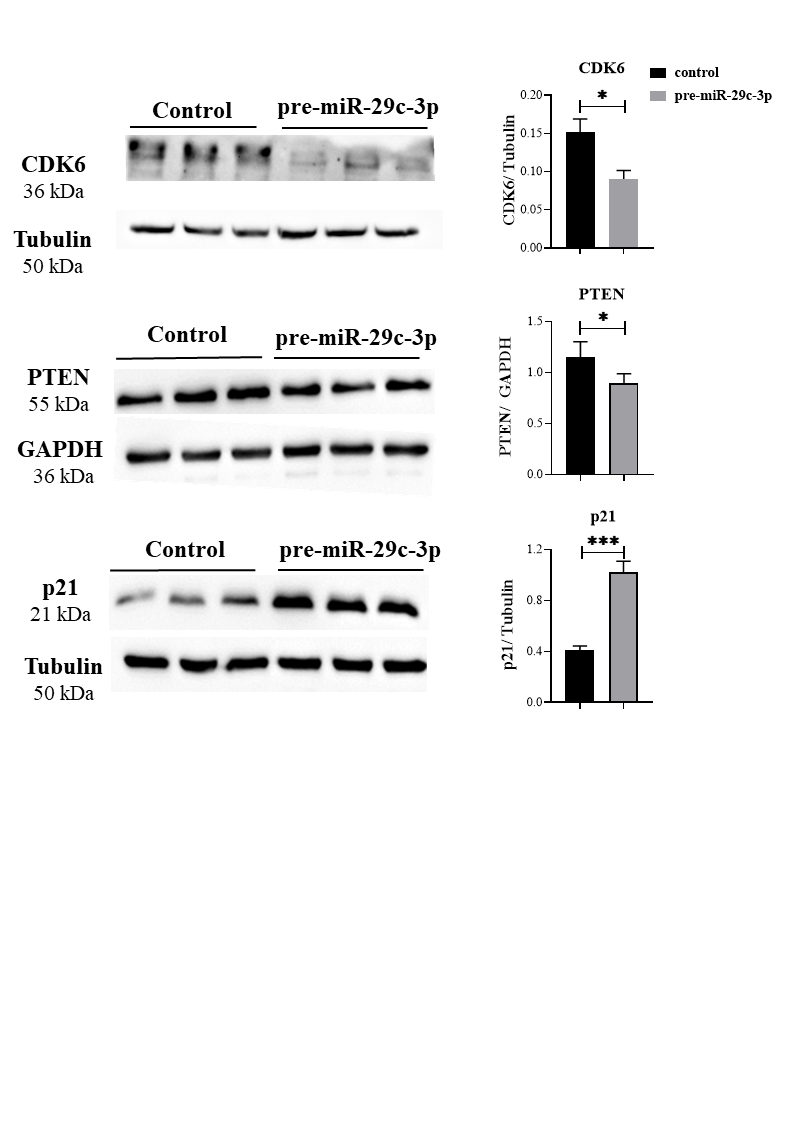

Supplement: Supplementary file 2 — Figure S2. [file RMB2-24-e12645-s001.tif]
